# Deep Vein Thrombosis-Induced Systemic Inflammation Triggering an Acute Polyarticular Gout Flare: A Case Report

**DOI:** 10.7759/cureus.95458

**Published:** 2025-10-26

**Authors:** Alex Abouafech, Daniel P Oar, Harrison Mahon, Joe Bhagratie

**Affiliations:** 1 School of Medicine, Lake Erie College of Osteopathic Medicine, Bradenton, USA; 2 Orthopedic Surgery, Lake Erie College of Osteopathic Medicine, Bradenton, USA; 3 Orthopedic Surgery, Baptist Medical Center Jacksonville, Jacksonville, USA; 4 Orthopedics, Baptist Medical Center Jacksonville, Jacksonville, USA

**Keywords:** acute gout, deep vein thrombosis (dvt), gout flare, systemic and vascular inflammation, venous thrombo-embolism

## Abstract

Deep vein thrombosis (DVT) and gout are traditionally regarded as distinct conditions, yet emerging evidence suggests important inflammatory and clinical connections. We present a 40-year-old man with a history of crystal-proven gout who developed a left gastrocnemius DVT after prolonged travel, followed shortly by a severe polyarticular gout flare. Laboratory studies revealed marked hyperinflammation (C-reactive protein peaking at 87.8 mg/L) and a new positive antinuclear antibody titer (1:320, homogeneous). Arthrocentesis confirmed monosodium urate crystals, while magnetic resonance imaging demonstrated synovitis, a cartilaginous loose body, and degenerative changes. Coordinated care among hematology, rheumatology, and orthopedics involved anticoagulation management, intra-articular corticosteroid injection, and tailored anti-inflammatory therapy. This case illustrates how thromboinflammatory cytokines and oxidative stress accompanying DVT may precipitate acute gout, even in the absence of marked hyperuricemia. Recognizing this interplay is essential for timely diagnosis, safe therapeutic decision-making in the setting of anticoagulation, and ongoing surveillance for autoimmune disease.

## Introduction

Gout and deep venous thrombosis (DVT) are two common conditions encountered in clinical practice, affecting approximately 12 million and 900,000 individuals in the United States, annually [1,2]. Traditionally, these diseases are viewed as separate, unrelated entities. However, recent evidence highlights important biological and clinical connections between these two pathologies [3]. Gout is an inflammatory arthritis caused by monosodium urate crystal deposition, typically triggered by hyperuricemia [4]. Beyond classic precipitants, including dietary purines, alcohol, dehydration, and rapid changes in serum urate, systemic inflammatory stressors and immobility are increasingly recognized as powerful triggers of acute gout flares. Episodes of surgery, infection, or hospitalization can destabilize urate deposits, induce leukocyte activation, and precipitate intense joint inflammation [5].

DVT, on the other hand, is a thromboinflammatory disorder characterized by venous stasis, endothelial activation, and hypercoagulability, with significant morbidity and mortality in affected patient populations. In addition to the well-recognized Virchow triad, thrombosis itself provokes an inflammatory cascade of events [6]. The developing thrombus releases inflammatory cytokines, recruiting neutrophils and monocytes while promoting oxidative stress. This cytokine-rich, pro-oxidative condition can lower urate solubility, enhance monosodium urate crystal formation, and put patients at a heightened risk for acute gout flares [6-8]. Pain and swelling from DVT can additionally enforce prolonged limb immobility, which further increases local hypoxia and impairs lymphatic clearance, which promotes local inflammation and acute gout flares. The acute-phase response during DVT may act as a general systemic stressor that precipitates gout flares in predisposed individuals, even when serum urate is not markedly elevated. Epidemiologic data may also suggest a bidirectional association between these conditions. Patients with confirmed acute gout flares have been found to carry a higher risk of incident venous thromboembolism, potentially due to chronic low-grade inflammation, endothelial dysfunction, and shared metabolic risk factors such as obesity and metabolic syndrome [3,6-8]. A large-scale population-based cohort study by Lingyi et al. found that the overall risks of venous thromboembolism, pulmonary embolism, and DVT were significantly increased both before and after gout diagnosis compared to the general population, highlighting the important relationship between these two distinct disease processes [8].

Management of concomitant DVT and gout presents significant challenges for clinicians, as therapeutic strategies for one condition may complicate treatment of the other [3,8,9]. Anticoagulation, the cornerstone of DVT management, increases the risk of bleeding with common anti-inflammatory agents such as nonsteroidal anti-inflammatory drugs (NSAIDs) and corticosteroids, which are frequently used to manage acute gout flares [9]. Colchicine, another mainstay in gout therapy, requires dose adjustment in patients with renal impairment, a population already at heightened risk for both gout and thrombosis [10]. These overlapping pharmacologic and pathophysiologic considerations necessitate a nuanced, individualized approach to minimize adverse outcomes while effectively treating both conditions.

In this report, we describe a 40-year-old man who developed a left lower extremity DVT after prolonged travel, followed shortly by a severe polyarticular gout flare in the left lower extremity. This patient exemplifies how thromboinflammation and immobility from DVT can potentially trigger acute gout attacks, and underscores the diagnostic and therapeutic complexity when thrombotic, crystalline, and autoimmune processes converge. With a more comprehensive understanding of the complex interplay between these unique diseases, practitioners will be better equipped to deliver optimal patient care. 

## Case presentation

**Figure 1:**
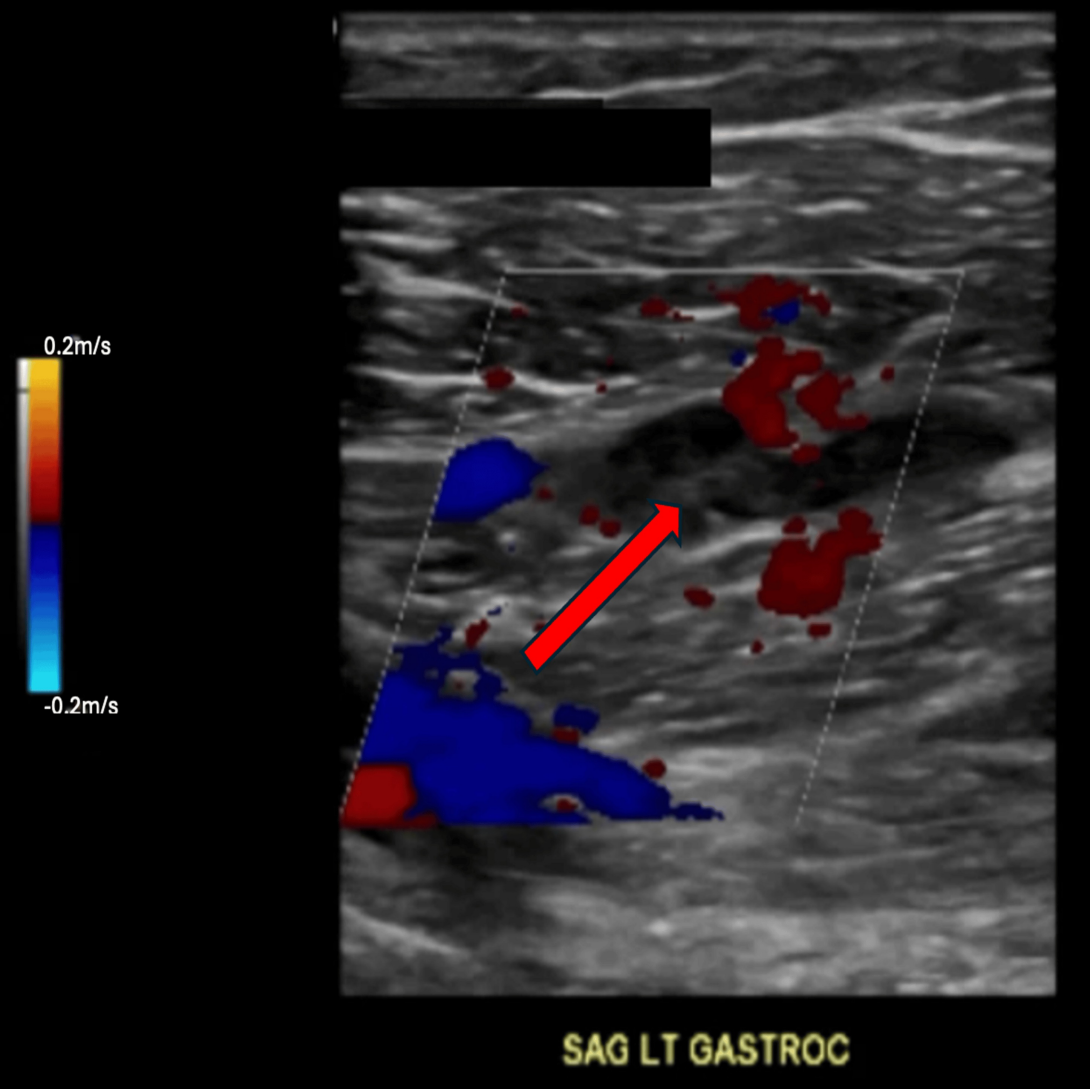
Ultrasound performed at initial presentation with sagittal view of left gastrocnemius vein showing a nonocclusive thrombus indicated by the red arrow demonstrating the echogenic material within the lumen with preserved compressibility and color flow around it. Red signal is blood flow towards the ultrasound probe and blue signal is blood flow away from the probe.

The patient is a 40-year-old man with a three-year history of crystal-proven gout. He is a nonsmoker, abstains from alcohol, and has no family history of thrombosis or autoimmune disease. He discontinued allopurinol in the past because of a rash and had intermittently taken febuxostat but stopped it recently. Following a long period of immobility during travel, the patient developed sudden swelling and pain of the left calf and knee. Venous duplex ultrasonography confirmed a short-segment nonocclusive deep venous thrombosis of the left gastrocnemius vein (Figure 1). Initial inflammatory markers were elevated, with C-reactive protein (CRP) 65 mg/L and erythrocyte sedimentation rate (ESR) 53 mm/h. He began oral anticoagulation with apixaban.

Three days later, the patient experienced progressive swelling and paresthesias of the left foot with worsening knee pain, prompting hospitalization. Neurology attributed the symptoms to compressive neuropathy. Blood cultures remained negative, and lumbar MRI showed only chronic degenerative disc bulges. CRP improved to 23.1 mg/L during hospitalization. Anticoagulation was transitioned to weight-based enoxaparin, 120 mg subcutaneous twice daily, and he was discharged on enoxaparin, gabapentin, 300 mg orally twice daily, and a brief course of doxycycline.

**Figure 2:**
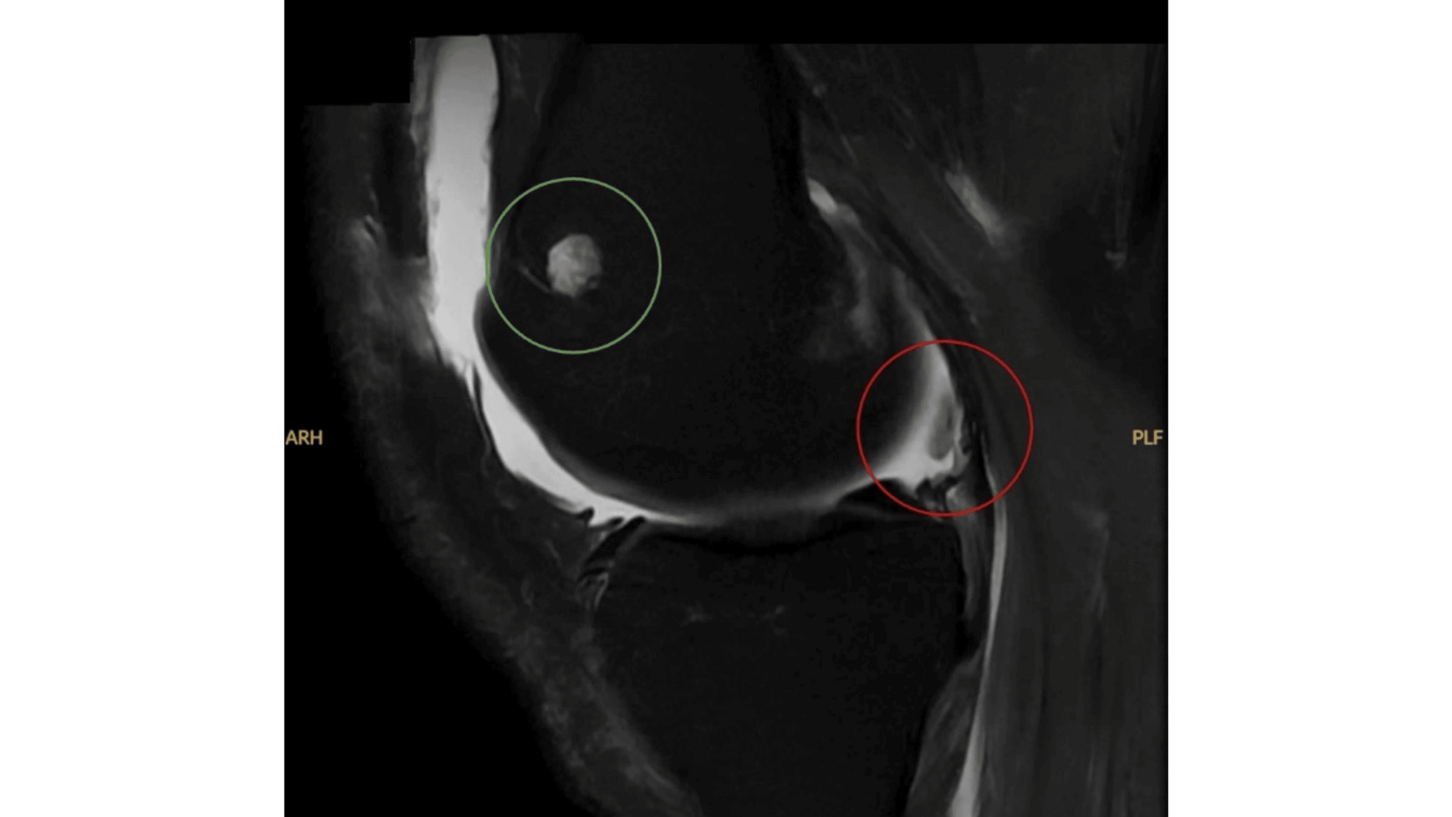
Sagittal T2-weighted MRI obtained during gout flare of the right knee shows moderate joint effusion and a cartilaginous loose body in the posterior and medial joint space measuring up to 14 mm (red circle) and a small benign enchondroma in the distal femur measuring 8 mm (green circle).

Soon after discharge, he developed new swelling and pain of the right knee and ankle, while the left knee remained symptomatic. Ten days removed from discharge, he reported intermittent fevers to 101 °F and worsening joint pain. Repeat laboratory evaluation showed a marked CRP rise to 87.8 mg/L, while blood cultures remained negative. Antinuclear antibody (ANA) testing was positive at a titer of 1:320 with a homogeneous pattern, raising concern for an evolving autoimmune process. However, no other autoimmune markers were tested (e.g., anti-dsDNA, extractable nuclear antigen (ENA)). Serum uric acid had previously been 5.0 mg/dL, but increased to 10.1 mg/dL during this flare. Lab values are summarized in Table 1. Arthrocentesis of the right knee with an anterior non-ultrasound-guided approach demonstrated monosodium urate crystals, along with cloudy synovial fluid confirming an acute gout flare. MRI of the right knee showed extensive synovitis with a 14 mm cartilaginous loose body in the posterior medial compartment (Figure 2). This intra-articular loose body was believed to result from intense crystal-driven inflammation causing chondral injury and fragmentation during the acute gout attack. Orthopedics performed an intra-articular corticosteroid injection and prescribed celecoxib, later switched to indomethacin. Because of the need for NSAID therapy and procedures, hematology supervised a brief interruption of enoxaparin.

**Table 1:** Summary of inflammatory markers prior to, during hospitalization, and at time of acute gout flare. "-"  missing value Serum uric acid reference range: 4.0 - 8.0 mg/dL

Test	Initial	During hospitalization	At gout flare
C-reactive protein (mg/L)	65 (mg/L)	23.1 (mg/L)	87.8 (mg/L)
Erythrocyte sedimentation rate (mm/hr)	53 (mm/hr)	-	63 (mm/hr)
Uric acid (mg/dL)	5.0 (mg/dL)	6.5 (mg/dL)	10.1 (mg/dL)
Antinuclear antibodies	-	-	Positive, 1:320, homogenous

At follow-up one month later, the patient's reported symptoms resolved with minimal residual right knee pain. The patient’s left knee pain and swelling had largely resolved, and his right knee continued to improve under colchicine, 0.6 mg taken once daily orally, and corticosteroid therapy, 4 mg pack dose. His kidney function remained stable with a blood urea nitrogen level of 16 mg/dL and a creatinine level of 0.84 mg/dL. He remained under rheumatology follow-up for possible connective tissue disease and hematology supervision for anticoagulation management.

## Discussion

This case highlights a clinically important and increasingly recognized intersection between venous thromboembolism and crystal-induced arthritis. The patient developed an acute polyarticular gout flare shortly after the onset of a left gastrocnemius deep venous thrombosis, suggesting that thromboinflammatory mechanisms can act as potent triggers of gout in susceptible individuals.

Venous thrombosis is now understood to be a highly inflammatory process rather than a purely mechanical event. Activated platelets and coagulation factors drive recruitment of neutrophils and monocytes, generating interleukin-6 (IL-6), tumor necrosis factor-α, and reactive oxygen species. Chronic inflammation accompanying these rheumatologic diseases is considered to increase the risk of venous thromboembolism by up-regulating procoagulants, down-regulating anti-coagulants, suppressing fibrinolysis, and causing endothelial dysfunction [11]. Monosodium urate crystals are pro-inflammatory stimuli that can initiate, amplify, and sustain an intense inflammatory response [11]. Rises in IL-6 and other cytokines amplify neutrophil recruitment and reactive oxygen species generation in tissues. In gout, these pathways facilitate neutrophil extracellular trap formations around monosodium urate and amplify joint inflammation [12]. These mechanisms provide a biologic explanation for how DVT can precipitate a clinically significant gout flare, even when serum urate is not dramatically elevated. Moreover, immobilization after DVT promotes cooler periarticular temperatures and relative hypoxia. Both of these factors lower urate solubility through temperature effects and lactic acidosis, which are known to further drive crystallization [13].

The overlapping presentation of thrombosis, gout, and possible autoimmune activity created a challenging diagnostic landscape. Fever, high C-reactive protein, and knee swelling prompted consideration of septic arthritis, meniscal injury, and evolving connective-tissue disease. Definitive demonstration of monosodium urate crystals on arthrocentesis established the primary diagnosis of acute gout, while the positive homogeneous antinuclear antibody titer raises the possibility of a latent autoimmune disorder such as systemic lupus erythematosus. Continued rheumatologic surveillance is warranted to determine whether the ANA reflects nonspecific inflammation or heralds a developing autoimmune condition.

Management required careful coordination among hematology, rheumatology, and orthopedics. Anticoagulation with low-molecular-weight heparin was essential for DVT, but it complicated the use of nonsteroidal anti-inflammatory drugs and intra-articular steroid injections-cornerstones of gout therapy-by increasing bleeding risk. Orthopedic input guided safe intra-articular corticosteroid administration and addressed concomitant structural pathology, including a cartilaginous loose body, which contributed to persistent knee pain. Rheumatology oversaw tailored anti-inflammatory therapy and planned for long-term urate-lowering strategies despite prior allopurinol intolerance.

Epidemiologic data support a bidirectional association between gout and venous thromboembolism. Patients with gout have higher rates of DVT and pulmonary embolism, while thrombotic events can trigger gout flares through systemic inflammation and immobility [7]. Recognizing this interplay has practical implications. Clinicians should maintain heightened vigilance for thromboembolic complications in patients with gout and, conversely, anticipate the possibility of gout flares during or shortly after thrombotic events or prolonged immobilization. Although a temporal relationship between DVT and gout flare was observed, future studies should investigate the bidirectional relationship between thrombosis and gout using prospective, cohort designs that measure changes in cytokines, oxidative stress markers, and serum urate during acute thrombotic events.

In summary, this case illustrates how acute DVT can precipitate a systemic gout flare via thromboinflammatory cytokines and oxidative stress. It underscores the need for early crystal confirmation, cautious anti-inflammatory therapy in the setting of anticoagulation, and ongoing surveillance for autoimmune disease. Appreciating these complex interconnections can improve diagnostic accuracy and guide safe, effective, multidisciplinary management.

## Conclusions

This case illustrates that inflammation from deep vein thrombosis can precipitate acute gout flares in predisposed individuals. Prompt confirmation of monosodium urate crystals is essential for accurate diagnosis, as overlapping symptoms may obscure the underlying cause. Management requires careful coordination between specialties to balance anti-inflammatory therapy with anticoagulation safety. Clinicians should remain vigilant for gout flares following DVT or other systemic inflammatory states to enable early recognition and targeted treatment. Further research into the bidirectional relationship between venous thromboembolism and gout may refine risk stratification and inform strategies to prevent recurrent events.
